# Transfer of more than two embryos, regardless of the age of the female partner, is not beneficial for neither the mothers nor the babies: lessons from the Latin American Registry of Assisted Reproductive Techniques

**DOI:** 10.5935/1518-0557.20170006

**Published:** 2017

**Authors:** Juan Enrique Schwarze, Javier Crosby

**Affiliations:** 1 Reproductive Medicine Unit at Clinica Monteblanco, Chile; 2 Obstetrics and Gynaecology Department at Universidad de Santiago, Chile; 3 Reproductive Medicine Unit at Clinica Las Condes, Chile

**Keywords:** embryo transfer, multiple delivery, ART, complications

## Abstract

**Objective:**

ART has helped millions of infertile couples worldwide to overcome their childlessness. These successes have been accompanied by an increase in multiple deliveries, and perinatal complications associated. The explanation for this complication is the transfer of more than one embryo, to increase the odds of delivery. Our objective was to compare the outcome of elective dual embryo transfer (eDET) to that of the transfer of more than two embryos without embryo cryopreservation (TET), terms of delivery rate and multiple delivery.

**Methods:**

We analyzed the data registered by 155 clinics members of the RLA: 11,024 eDET and 10,634 TET.

**Results:**

The delivery rate was significantly higher when eDET was performed than when TET was performed (40.24% and 26.98%, *p* < 0.001). Also, the ratio of twin deliveries was higher in eDET (25.80% and 20.56%, *p* < 0.001). However, the ratio of triplets and more deliveries was higher in TET than in eDET (2.34%and 0.52%, *p* < 0.001). These findings were consistent across the different age categories of the female partner.

**Conclusion:**

Our findings suggest that eDET was associated with a statistically significant better delivery rate per embryo transfer, and lower ratio of triplet-and-higher deliveries, regardless of the woman’s age. Therefore, there is no evidence that supports the transfer of more than two embryos.

## INTRODUCTION

Between 1990 and 2013, 144,287 babies have been born in Latin America due to assisted reproductive techniques. These success stories have been associated with an increase in multiple births because of the transfer of more than one embryo. Indeed, according to the latest 2013 report, more than two embryos were transferred in 25,9% fresh autologous IVF/ICSI embryo transfers. Furthermore, 20.71% of deliveries corresponded to twins and 1.09% to triplets and higher number of babies (Zegers-Hochschild *et al*., 2016).

Multiple pregnancies and deliveries have been associated with an increase in the risk for the mother (gestational hypertension, preeclampsia, gestational diabetes) and for the neonate (preterm delivery, low birth weight (Qin *et al*., 2015; Geisler *et al*., 2014).

Although it is widely recognized that elective single embryo transfer (eSET) is the only way to avoid multiple pregnancies and multiple births, only 1.4% of embryo transfers performed in the region corresponded to eSET, while 21% corresponded to the elective transfer of two embryos (eDET) (Zegers-Hochschild *et al*., 2016). Furthermore, the transfer of ≥ 3 embryos corresponded to 25,9% of all embryo transfers, a ratio that increases to 26.8% in women aged 35 to 39 years; 33.23% in women aged 40 to 42 years old; and 33.18% in women aged ≥ 43 years old.

Several barriers prevent the implementation of a policy of electively transferring less embryos. Patients and clinicians may be unwilling to neither accept nor offer the transfer of less embryos, as a result of the expected lower likelihood of pregnancy or live birth rates, specially so in women over 40 years of age; especially when the treatment-associated costs are covered by the patient's own money, as is the case in the vast majority of Latin American couples (Chambers *et al*., 2014).

Our objective was to assess the outcome of eDET concerning the transfer of more than two embryos in different woman´s age category in terms of success (delivery rate) and complications (multiple delivery).

## MATERIAL AND METHODS

The Latin American Registry of ART keeps a database of a case-by-case registry, that keeps track of individual data from controlled ovarian hyperstimulation protocols until the birth of the neonate(s). All informed consent forms state that the data can be used, anonymously, for epidemiological studies. Given these, no other consent form was requested for the purposes of this study.

Biomedical data of fresh IVF/ICSI embryo transfers were extracted from oocyte retrievals between January 1^st^, 2012 and December 31^st^, 2013. This data included: age of female partner in completed years, stage of embryo development upon embryo transfer (cleaving embryo or blastocyst), number of babies born (singletons, twins, triplets and more), gestational age in completed weeks of amenorrhea, perinatal viability, and birth weight in grams. We used ICMART´s revised glossary of ART terminology (Zegers-Hochschild *et al*., 2006).

We compared the outcome of two-embryo transfer policies – regardless of embryo development at embryo transfer: when two embryos were transferred and at least one embryo was cryopreserved (eDET), and when three or more embryos were transferred and no embryo was cryopreserved (TET). The outcomes analyzed were: delivery rate per embryo transfer and multiple deliveries at different age categories: ≤ 34 years old, 35-39 years old, 40-42 years old, and ≥ 43 years old.

We used Fisher´s exact test to test for independence of association. To assess for normal distribution, we used the Shapiro-Wilk test. In the case of non-normally distributed variables, we compared the mean values using the Mann-Whitney U test. When appropriate, we present the relative risk (RR) with its correspondent 95% confidence interval (95%CI). We performed a logistic regression analysis, adjusting for maternal age in completed years to embryo development at the time of transfer (blastocyst stage and cleaving-embryo), to compare the effects of eDET and TET on the odds of delivery per embryo transfer, and the odds for triple-and-higher deliveries. A p-value lower than 0.05, was considered statistically significant. All statistical analyses were performed using STATA (Statcorp, USA).

## RESULTS

In the study period, 155 clinics members of the RLA reported a total of 68,351 initiated IVF/ICSI cycles and 49,637 embryo transfers; 11,024 corresponded to eDET and 10,634 to TET.

The mean age of the female partner was significantly higher in the TET group. The ratio of blastocyst-stage at embryo transfer was higher in the eDET group (Table 1).

**Table 1:** Characteristics of cycles analyzed, IVF/ICSI 2012-2013

	eDET	TET	*p*-value
Number of transfer cycles	11002	10634	
Mean age of female partner (SD)	34.07 (4.09)	37.32 (4.21)	< 0.001
Proportion of blastocyst-stage transfer	30.96%	11.49%	< 0.001
Delivery rate per embryo transfer	40.24%	26.98%	< 0.001
Deliveries (n)	4427	2869	< 0.001
Singletons (%)	73.68	77.10	
Twins (%)	25.80	20.56	
≥ Triplets (%)	0.52	2.34	
All newborns			
mean weight g (SD)	2698 (666)	2645 (655)	0.0024
mean gestational age WA (SD)	36.9 (2.7)	36.8 (2.5)	0.0019
perinatal mortality ‰	36.0	45.9	0.017
Singletons			
mean weight g (SD)	3050 (534)	2968 (502)	< 0.001
mean gestational age WA (SD)	38 (2.2)	37 (2.1)	< .001
perinatal mortality ‰	31.6	36.2	0.354
Twins			
mean weight g (SD)	2265(515)	2243 (522)	0.2413
mean gestational age WA (SD)	35 (2.9)	35 (2.7)	0.1501
perinatal mortality ‰	41.6	53.4	0.115
≥ Triplets			
mean weight g (SD)	1579 (472)	1689 (448)	0.0766
mean gestational age WA (SD)	32 (2.9)	33 (3.0)	0.1814
perinatal mortality ‰	60.6	102.6	0.308

eDET transfer of two embryos and at least one embryos cryopreserved; TET transfer of at least three embryos and no embryo cryopreserved; g = grams; WA = weeks in amenorrhea; SD standard deviation; Perinatal mortality: fetal or neonatal death occurring during late pregnancy (at 20 completed weeks of gestational age and later), during childbirth, or up to 7 completed days after birth.

The delivery rate per embryo transfer was significantly higher when eDET was performed than when TET was performed (40.24% and 26.98%, *p* < 0.001). Also, the ratio of twin deliveries was higher in eDET than in TET (25.80% and 20.56%, *p* < 0.001), while the ratio of triplets-and-higher order deliveries was lower (0.52% and 2.34%, *p* < 0.001; Table1).

Table 1 also shows mean neonatal weight, mean gestational age at delivery, and perinatal mortality per gestational order. In the case of singletons, the mean neonatal weight, and mean gestational age were higher in the eDET group, whereas perinatal mortality was lower. In the case of twins and ≥ triplets, we did not find a significant difference in these variables.

In all age categories analyzed, the delivery rate per embryo transfer was significantly higher in the eDET group (Table 2). The mean number of embryos transferred in the TET group did not vary significantly with woman´s age category: it was 3.06 (range 3-6) in women ≤ 34 years, 3.06 (range 3-5) in women 35 to 39 years, 3.17 (range 3-5) in women 40-42 years, and 3.25 (range 3-6) in women ≥ 43 years. We performed a logistic regression to establish the effects of eDET over TET on delivery rate per embryo transfer. After correcting for age of woman and embryonic development at embryo transfer, we found that eDET had an OR for delivery rate per embryo transfer of 1.38 (95% CI 1.30-1.47 *p* < 0.001).

**Table 2:** Comparison of delivery rate per embryo transfer according to woman´s age category between eDET and TET, IVF/ICSI 2012-2013

Age category	eDET		TET		RR (CI 95%)
	Embryo transfers	Delivery rate (%)	Embryo transfers	Delivery rate (%)	
≤ 34	5295	45.04	2174	35.51	1.27 (1.19-1.35)
35-39	4454	39.25	4459	30.70	1.28 (1.21-1.35)
40-42	1030	24.17	2788	20.27	1.19 (1.04-1.36)
≥ 43	223	20.18	1213	13.44	1.5 (1.12-2.02)
TOTAL	11002	40.24	10634	26.98	1.49 (1.43-1.55)

eDET transfer of two embryos and at least one cryopreserved embryo; TET transfer of at least three embryos and no cryopreserved embryo; RR = relative risk; CI 95% = 95% confidence interval

Overall, 25.80% of deliveries in the eDET group were twins, whereas 20.56% of deliveries in the TET group were twins (*p* < 0.001), as seen on Table 3. In the case of eDET, when cleaving-stage embryos were transferred, the ratio of twin deliveries was 22.06%, when blastocyst-stage embryos were transferred, the ratio of twin deliveries increased to 32.63%

**Table 3:** Comparison of gestational order according to woman´s age category between eDET and TET, IVF/ ICSI 2012-2013

Age category	eDET				TET				RR for triplet delivery (CI 95%)
	Number of deliveries	Singletons (%)	Twins (%)	Triplets (%)	Number of deliveries	Singletons (%)	Twins (%)	Triplets (%)	
≤ 34	2385	71.15	28.26	0.59	772	69.69	25.91	4.40	0.38(0.25-0.59)
35-39	1748	75.06	24.43	0.52	1369	77.21	21.11	1.68	0.50(0.29-0.87)
40-42	249	85.94	14.06	0.00	565	84.42	14.16	1.42	NA
≥ 43	45	86.67	13.33	0.00	163	85.89	12.88	1.23	NA
Total	4427	73.68	25.80	0.52	2869	77.10	20.56	2.34	0.42 (0.29-0.69)

eDET transfer of two embryos and at least one cryopreserved embryo; TET transfer of at least three embryos and no cryopreserved embryo; RR = relative risk; CI 95% = 95% confidence interval; NA = not applicable.

The ratio of triplet-and-higher deliveries was higher in the TET group, regardless of age category. Overall, 0.52% of deliveries in the eDET group were triplet-and-higher, whereas 2.34% of deliveries in the TET group were triplet-and-higher (*p* < 0.001) (Table 3). We performed a logistic regression analysis to determine the effect of eDET over TET on triplet-and-higher delivery rates. After correcting for age of woman and embryonic development at embryo transfer, we found that eDET had an OR for triplet-and-higher deliveries of 0.14 (95% CI 0.08-0.23, *p* < 0.001).

Furthermore, we analyzed perinatal mortality after eDET and TET. As expected, it was higher after TET (46.9‰) than after eDET (36.0‰), explained partially by the higher triplet-and-higher deliveries. However, among singletons, perinatal mortality was also higher after TET than after eDET (36.2‰ and 31.6‰, respectively).

## DISCUSSION

In the present study, with 11,002 eDET and 10,634 TET, we found that regardless of woman´s age, eDET was associated with a significantly better delivery rate per embryo transfer, and a significantly lower ratio of triplet-and-higher delivery.

Our study has two main advantages. First, our database corresponds to a case by case registry, thus it contains information of every single case, allowing for a more thorough biostatistical analysis. Second, it corresponds to a multi-centric and multinational database, which allows for external generalization.

The main disadvantage of our study is that it corresponds to an observational study, not a randomized controlled study, thus caution must be exerted when analyzing the data and causation attributed. However, we corrected for known confounding variables such as maternal age and embryonic development at embryo transfer. Nevertheless, there might be unknown factors that only can be accounted for by means of randomization to eDET or TET.

It is certainly surprising that TET was associated with a significantly lower delivery rate per embryo transfer, in all age categories. We hypothesize that this might be explained by a negative selection bias, in which poorer-prognosis cases are detected through embryo development and clinical criteria associated to transferring more embryos. As a matter of fact, the mean age of women undergoing TET was significantly higher than that of women undergoing eDET; nevertheless, after correcting for woman´s age, eDET was still significantly associated with a better prognosis. The only means to address this theoretical bias would be by randomizing the number of embryos to be transferred. Regardless of this possible bias, we found that TET was associated with a statistically significant increased risk of triple-and-higher delivery rates. Which in term, is associated with an increase in preterm birth and perinatal mortality. Interestingly, even in the case of singleton deliveries, TET was associated with an increase in perinatal mortality, without reaching statistical significance.

Our results differ to that published by Dare *et al*. (2004). They compared the outcome of double embryo transfer with that of three-or-more embryo transfer. They found that the transfer of two embryos was associated with a lower incidence of live birth at term, clinical pregnancy, multiple pregnancy and multiple birth, twin birth, triplet or higher order pregnancy, and triplet or higher order births (Dare *et al*., 2004). This difference might be explained by the improvement in technology during the last ten years. However, most of the literature deals with the outcomes of transferring fewer embryos in good-prognosis patients. Kissin *et al*. (2014) published, in their population-based study, the association between number of embryos transferred and good perinatal outcome by age, embryo stage, and prognosis. According to their analysis they concluded that among patients younger than 35 years of age undergoing *in vitro* fertilization with a favorable prognosis, the highest chance of good perinatal outcome is associated with single embryo transfers. In the latest Cochrane review, the authors concluded that in a single fresh IVF cycle, single embryo transfer is associated with a lower live birth rate than double embryo transfer. However, there is no evidence of a significant difference in the cumulative live birth rate, and single embryo transfer is associated with much lower rates of multiple pregnancies. Furthermore, they pointed out, that most of the evidence currently available concerns younger women with a good prognosis (Pandian *et al*., 2013).

As stated previously, a possible barrier for patients and physicians to transfer less embryos, is the perceived minor likelihood of delivery, when less embryos are transferred, especially so in women with poorer prognosis e.g. older women. Our analysis shows that this is not the case; eDET was associated with a statistically significant better delivery rate per embryo transfer, and lower ratio of triplet-and-higher deliveries. We hope that this study and others will help both physicians and infertile couples to make better informed choices.
